# Ethnomedicinal Uses of Honeybee Products in Lithuania: The First Analysis of Archival Sources

**DOI:** 10.1155/2016/9272635

**Published:** 2016-08-25

**Authors:** Zivile Pranskuniene, Jurga Bernatoniene, Zenona Simaitiene, Andrius Pranskunas, Tauras Mekas

**Affiliations:** ^1^Department of Drug Technology and Social Pharmacy, Lithuanian University of Health Sciences, Kaunas, Lithuania; ^2^Museum of the History of Lithuanian Medicine and Pharmacy, Kaunas, Lithuania; ^3^Department of Intensive Care Medicine, Lithuanian University of Health Sciences, Kaunas, Lithuania

## Abstract

Lithuania has old ethnomedicine traditions, consisting of many recipes with herbal, animal, and mineral original ingredients. All these findings were mostly collected in Lithuanian language, often in local community's dialects, and stored only in archives. We analyzed archival sources about honeybee and its products used for medicinal purposes dated from 1886 till 1992 in different parts of Lithuania. We systematized and presented the most important information about bees and their products: indication for usage, ingredients used in the recipe, their preparation techniques, and application for therapeutic purposes. Researchers in Lithuania are now looking for new evidence based indications and preparation and standardization methods of bee products. Archival sources are a foundation for studies in Lithuania. The results can be integrated into scientifically approved folk medicine practices into today's healthcare.

## 1. Introduction

The first knowledge about Lithuanian ethnomedicine is mentioned in the chronicles of the Teutonic Knights about spells, prejudice, and traditions, since they have close connection to the folk medicine. There are documents about ethnomedicine in the 16th-17th centuries' law books of Grand Duchy of Lithuania. Cases of witches courts with witchcraft methods were mentioned. Lithuania became Christian country latest in Europe, while pagan traditions still existed after christening for a long time. These traditions had close connection with ethnomedicine [1, 2].

The biggest part of all Lithuanian ethnomedicine consists of the usage of traditional medicinal plants. Ethnobotanical expeditions were organized in various historical periods with the aim of preserving local knowledge about traditional uses of medicinal plants for medicinal purposes. All these findings were mostly collected in Lithuanian language, often in local community's dialects, and stored only in archives [3].

Many countries have recently engaged into studies of traditional medicine [4, 5] and Lithuanian researchers begin to collect, systematize, analyze, and publish ethnomedicinal studies as well [6–8]. According to ethnomedicinal global trend studies, it is important to find analysis of food on one hand and medications on the other. These categorizations offered possibility of generating information that could be ordered in a similar way in studies from different regions of the world and thus helped to make comparisons between them [9].

Ethnomedicinal preparations of animal origin make up smaller part of preparations; however, usage of preparations of animal origin is very important part of Lithuanian ethnomedicine. Bee products were important not only for nutritional purposes but also for their healing features and wide medicinal application [10–12]. Bee products can be used alone or in combination with medicinal plants, substances of animal origin, or strongly acting materials for synergistic effect, cumulative impact, or just better taste and administration form. In addition to bee products as food, active substances used for medicinal purposes, and inactive substances used as bases (e.g., as ointment base), they show close relationship between animal and herbal origin substances used in homemade medicine. This is a part of traditional ecological knowledge, which represents a close relationship between people and places [13].

To find parallels within ethnopharmaceutical (including bee products) research, we look to countries with similar history, nature, and ethnomedical traditions. For this reason, in most cases, Lithuanian researches compare their studies with studies from Poland. Unfortunately, such works are limited [3, 14]. Information about medicinal plants and animals traditionally used for therapeutic purposes is mainly deficient, since archive material is not systematized and mostly presented in small ethnographic papers published in native languages [15, 16]. This problem, also with scarce information on bee products, highlights Polish researches [3, 6, 17]. Also researches organize studies with migrant communities and investigate situation; then people moved to urbanized areas and despite the availability of primary healthcare they often bring with them the traditional medical knowledge and actively use it. Systematic archival material can facilitate comparative analysis in such kind of investigations [18, 19].

Purpose of our study was to systematize unpublished archival material which concerns the use of bee products from ethnographic expeditions in Lithuania. This is the first analysis of unpublished material not only for scientific audience but also in local literature too. This study is important for complementing Lithuanian ethnomedicine data base and also can be useful for researches from other countries to find parallels between studies. Recently, researchers in Lithuania are looking for new evidence based indications and preparation and standardization methods of bee products. Certainly, ideas come from Lithuanian ethnomedicine archives and nowadays public opinion research.

## 2. Methods

We analyzed archival sources, that is, the material from ethnographic expeditions, dated from 1886 till 1992 in different parts of Lithuania. Among plenty of ethnobotanical information, it also includes preparations from animal origin. We systematized and presented the most important information about bee and its products: indication to use, ingredients used in the recipe, their preparation techniques, and application for therapeutic purposes.

## 3. Results and Discussion

In Lithuanian ethnomedicine, we can find recipes with bee products, and even whole bee, for the treatment of various ailments. We registered 65 reports regarding bee products used for medicinal purposes (Table 1). Only 39% of all usage was for internal use and the most popular preparation methods were ointments and compresses (22% and 19% from all reports, resp.). The most popular indications were abscesses, wounds, and contagious diseases, such as measles and smallpox. These explain bee products as antimicrobic substances. Honey is the only one of our registered bee products used internally (despite some cases of whole bee, honeycomb, and propolis) and it is the most popular from registered products.

Scientifically inexplicable usage of bee products is explained by archaic traditions and observation of the natural world and an understanding based on the theory of signatures. Only in this way can we explain bee usage as an antidote from viper's bite and fumigation with honeycombs for the same indication.

We registered cases when bee products are used alone or in combination with plants, animals, or additional material: 11 plant species and 8 animal products, such as powders of dried toad, swine bile, goose, chicken fats, dog's and pig's lard, cow's milk, and chicken's eggs. As additional products, cream, butter, vodka, and soap material were used in household.

### 3.1. Bee, Apes

Until the 18th century bee was described as diuretic and hair loss inhibitive remedy in pharmaceutical literature [20]. It was used in homeopathy for the treatment of allergy and swellings. In Lithuanian folk medicine, according to archival sources, bee's poison was used as antidote to viper's poison. “For epilepsy healing, the drinking the water with boiled dead bees (dead after the winter time) were used” (Table 1). Numerous practices used whole bee for medicinal purposes: “bee glue was used to put on ulcers and boils”; “bee glue was used to put on purulent gatherings for faster removing of purulence” [archival source: LLTI, 1081, pp. 55].

### 3.2. Honey, Mel

In Old Egypt, in the Ebers Papyrus, honey was the only active ingredient in an ointment described for application to the surgical wound of circumcision [5]. Honey could provide some kind of protection from various kinds of bacteria. It was used on the infected wounds to encourage the healing processes. The ancient Egyptians were not the only people who used honey as medicine. The Chinese, Indians, Ancient Greeks, Romans, and Arabs used honey in combination with other herbs or on its own to treat wounds and various other diseases [21].

Honey is one of the oldest and most famous materials of animal origin. There was a big amount of external drug forms containing honey in Lithuania. According to archival sources, honey (for external usage, alone or in combination) was usually used to treat wounds, abscess (Table 1), and even pain in the body: “from the pain in the body I have mix honey, butter, fat and rue leaves and used a teaspoon daily. After one week my health improved and pain disappeared” [archival source: LLTI. B4575.733/117]. In the Polish traditional medicine, honey has been used for respiratory diseases, gastrointestinal disorders, and dermatological problems. According to our study, the most popular indication for internal use of honey was respiratory disorders and no reports for gastrointestinal disorders were mentioned. But today's study with Polish settlements in Argentina demonstrates that most popular indication is also respiratory disorders [9]. Honey is the most popular material of bee products used up to now according to ethnomedicine studies in other countries and researches mainly focus on these studies [22, 23].

Nowadays, scientific studies indicate that honey contains major amounts of carbohydrates, lipids, amino acids, proteins, vitamins, and minerals that have important roles in wound healing with minimum trauma during redressing. Laboratory studies and clinical trials have shown that honey promotes autolytic debridement, stimulates growth of wound tissues, and stimulates anti-inflammatory activities, thus accelerating the wound healing processes [24]. Internally, it was used for various respiratory tract disorders. When ingested, honey also promotes healing and shows antibacterial action by decreasing prostaglandin levels, elevating nitric oxide levels, and exerting prebiotic effects. The use of honey leads to improved wound healing in acute cases, pain relief in burned patients, and decreased inflammatory response in such patients [25, 26].

According to the scientific studies, honey has antiseptic, curative properties and acts as effective broad-spectrum antibacterial agent [26]. The antimicrobial qualities of honey explain the external and internal uses of honey in Lithuania.

### 3.3. Bee Stings, Venenum Apium

Bee stings are some kind of injections and were used for treating rheumatism (Table 1). According to archival material, “bee stings were used for arthritis treatment”; “bee stings were used for rheumatizm healing” [archival source: LLTI, 2574, pp. 13].

Other researches highlight the therapeutic application of bee venom which has been used in traditional medicine to treat diseases, such as arthritis and rheumatism, and to relieve pain [27] and clinical trials also reveal bee sting therapy for rheumatoid arthritis and get positive results [28]; also bee venom acupuncture for rheumatoid arthritis is one of the opportunities for the treatment [29].

After viper's bite, according to archival sources, “just let bee to sting or put bee into bread and give it to eat” [archival source: LLTI, 3503, pp. 84].

### 3.4. Propolis, Propolim

Dioskorid described propolis as sliver extractor and also for fumigation from chronic cough. This is a pharmacopoeial remedy from the 16th century to the 18th century [20]. In Lithuania, it was used as oily or alcohol extract. According to our archival sources, propolis was used to treat wounds and joint pain. It is prepared with ethanol or with oily solution (Table 1).

Despite recent advances in wound care products, traditional therapies based on natural origin compounds, such as plant extracts, honey, and propolis, are interesting alternatives. These therapies offer new possibilities for the treatment of skin diseases and allow overcoming some limitations such as the increase in the bacterial resistance. Current trends move to the development of innovative wound care treatments, combining the use of traditional healing agents (such as propolis and honey) and modern products, such as dressing films and hydrogel sheets containing honey [30]. Also studies show that propolis is a potent antioxidant and a free radical scavenger [31, 32].

Lithuanian scientists have been focusing on investigation of propolis qualities and propolis preparations development. They identified that propolis therapeutic application does not induce germ resistance and does not destroy useful microflora [33]; study explains our findings using propolis for wound care not only with ethanolic solution, but also in a form of oily solution (Table 1). Nowadays, bee products, particularly honey and propolis and its preparations (tablets, suppositories, ointments, mouth sprays, and others), are available in most of the Lithuanian community pharmacies usually positioned as dietary supplements [34].

### 3.5. Beeswax and Honeycomb, Cera Alba, Flava, and Favum Mellis

Fumigation with Ibio shaped piece of wax was described in Ebers Papyrus. It was used when the uterus went down. Dioscorides described the pills of beeswax as a remedy that stops diarrhea [20].

Fumigation with wild honeycombs is very old method of cure, used to cure viper's bite (Table 1). Also, “if children got a fright, the honeycomb tea was used for treating” [LLTI number 6160.81].

White beeswax and yellow beeswax were an important part of ointments and plasters. Other researches investigating ethnopharmaceutical formulations in other countries also find beeswax as material for formulation of ointments [35]. In a study done by Kacániová et al. [36], it was found that the extracts of beeswax were effective against pathogenic bacteria, so this material can be used as antimicrobial agent too. It explains our findings for beeswax used in ointments form to treat wounds, abscesses, and burned skin.

**Figure 1:**
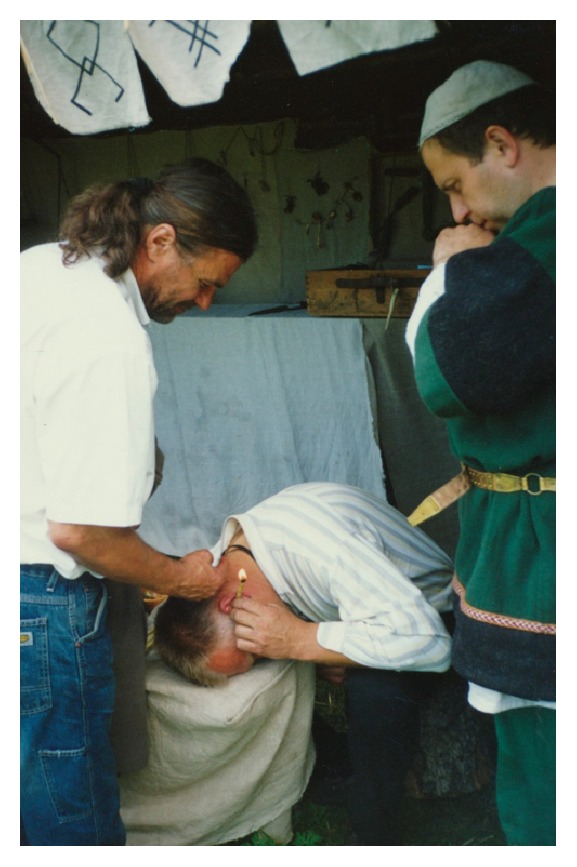
Ear pain relief by burning of rolled waxy linen cloth (demonstration).

In Lithuanian ethnomedicine, ear pain relief by burning of rolled waxy linen cloth is very interesting (Figure 1). The same method is known in Chinese medicine but instead of linen silk is used. Also beeswax still is used as a component in cosmetic preparations (ointments, lip pencils, etc.), for its acting as protective film on the skin and mucous membranes [37].

## 4. Conclusions

Treatment techniques with bee and its products in Lithuanian ethnomedicine have survived since the times when qualified medical assistance was hardly accessible. It is a unique fact that in modern times of developed medical assistance even young people in Lithuania actively use traditional bee products and combine them with modern medicine. These unpublished archival materials demonstrated that bee products not only were a part of plant or animal origin homemade medicines but also were among main ingredients in the recipes for the treatment and prevention of common diseases in the studied area. Archival sources are a foundation for studies in Lithuania.

The results can be integrated into scientifically approved folk medicine practices into today's healthcare.

## Figures and Tables

**Table 1:** Honeybee and its products used for medicinal purposes in Lithuania.

Bee and its products	Usage	Indication	Ingredients	Preparation method	Usage method	Archival source number
Bee, *Apes*	External	Viper's bite	Whole bee	Compress	Application to poisoned part	MT T3, P. 118–120
	External	Toothache	Whole dead bee	Decoction	Rinse the painful part	LLTI BK III-1167
	Internal	Viper's bite	Whole bee	Live bee	Oral	MT T3, P. 118–120; MMNB F127-87, P. 2–4
	Internal	Swollen throat	Whole bee	Dried bee	Oral	MT T5, P. 19–25
	Internal	Epilepsy	Whole bee	Decoction of dried bees	Oral	LNMA F.NM B75, P. 12; VUBRS F81-115, P. 54-55

Honeycomb	External	Viper's bite	Whole honeycomb		Fumigation	MT T3, P. 118–120
	Internal	Children's startle	Whole honeycomb	Decoction	Oral	LKDA B6160, P. 19–81

Honey, *Mel communis*	External	Tetter	Whole	Ointment	Applied to damaged part	LLTI BK III-653
	External	Neck pain	Whole	Ointment	Applied to damaged part	MT T5, P. 198
	External	Tonsillitis	Whole	Ointment	Applied to damaged part	LMDA I 211 a./20
	External	Joint pain	Whole	Ointment	Applied to damaged part and heated in the sun	IIES 208, P. 872
	External	Abscess	Whole	Ointment	Applied to damaged part	LLTI BK III-901
	External	Tonsillitis	Honey, water	Decoction	Rinse the painful part	MMNB F 25-188, P. 45
	External	Abscess	Honey, backfat	Compress	Application to damaged part	VUBRS F81-92, P. 30; LKDA B642/5, P. 58
	External	Pneumonia	Honey, *Nicotiana tabacum *L.	Compress	Application to the breast	VUBRS F81-92, P. 21
	External	Abscess	Honey, powder of dried toad	Compress	Application to damaged part	LLTI B2574, P. 18
	External	Abscess	Honey, tuber of *Allium sativum *L.	Compress	Application to damaged part	LLTI KN.605, P. 193–195
	External	Wound	Honey, *Arctostaphylos uva-ursi *(L.) Spreng.	Compress	Application to damaged part	LMDA I 283/44921
	External	Slag	Honey, milk	Compress	Application to damaged part	VUBRS F81-92, P. 33
	External	Tonsillitis	Honey, dried toad powders	Compress	Application to damaged part	LNMA KN 270, P. 477
	External	Bone pain	Honey, *Nicotiana tabacum *L.	Compress	Application to damaged part	LKDA B642/5, P. 58
	External	Hernia	Honey, swine bile	Compress	Application to damaged part	MMNB F25-188
	External	Abscess	Honey, goose fat	Compress	Application to damaged part	IIES 208/192, P. 882
	External	Wounds	Honey, tuber of *Allium cepa *L., cream, butter	Ointment	Application to damaged part	LMM, P. 171
	External	Wounds	Honey, beeswax, chicken fat, butter	Ointment	Application to damaged part	LMM, P. 155
	External	Wounds, abscess	Honey, decoction of *Matricaria recutita* L. flowers, juice of *Aloe vera *(L.) Burm.f., melted butter	Ointment	Application to damaged part	MMA A424, P. 36-37
	External	Infected wound	Honey, beeswax, fir or pine resin, tuber of *Allium sativum *L*., *melted butter or suet, juice of *Aloe vera *(L.) Burm.f.	Compress with linen cloth	Application to damaged part	MMNB F 127-87, P. 35–37
	External	Eyestrain	Wild bee's honey	Raw	Application to damaged part	LMDA I 211 a./26
	Internal	Measles	Honey	Raw	Oral	MMNB F 127-87, P. 35–37
	Internal	Wounds	Honey	Raw	Oral	LLTI B 2750, P. 326
	Internal	Eyestrain	Wild bee's honey	Raw		LMM, P. 155
	Internal	Cough	Honey, leaves of *Aloe vera *(L.) Burm.f.	Maceration	Oral	LMM, P. 10
	Internal	Phthisis, tonsillitis	Honey, juice of *Aloe vera *(L.) Burm.f.	Maceration	Oral	MMNB PR.957, P. 2
	Internal	High blood pressure	Honey, dried herb of *Tanacetum vulgare* L.	Maceration	Oral	MMA A418, P. 2
	Internal	Smallpox	Honey, vodka	Extraction	Oral	MMNB 544
	Internal	Tonsillitis	Honey, *Zingiber officinale* Roscoe	Extraction	Oral	MMNB F127-87, P. 5
	Internal	High blood pressure	Honey,* Anethum graveolens *L.	Maceration	Oral	MMA A418, P. 2
	Internal	Bronchitis	Honey, juice of *Viburnum opulus *L. berries, juice of *Beta vulgaris* L.	Maceration	Oral	IIES 208/191, P. 876
	Internal	Cough, phthisis, sore throat, dyspnea	Honey, hot milk		Oral	LLTI BK III-451; LLTI B4260, P. 63/210
	Internal	Scarlatina	Honey, dried toad powders	Decoction	Oral	LLTI B4258, P. 3; LLTI B2574, P. 6;. 63.
	Internal	Tonsillitis	Honey, ashes of toad		Oral	MMA A418, P. 1
	Internal	Cough	Honey, tuber of *Allium sativum *L.		Oral	LNMA F NM B75, P. 4
	Internal	Phthisis	Honey, dog's lard, butter		Oral	LLTI B4258, P. 22-23
	Internal	Rheumatism	Honey, ants	Decoction	Oral	MMA A418, P. 1

Bee stings	External	Rheumatism, arthritis	Bee stings	Raw	Application to damaged part	LLTI B4258, P. 18

Propolis	External	Abscess	Propolis	Raw	Application to damaged part	LMDA I 1060/412; LLTI B1081, P. 55

Propolis	External	Joints pain	Propolis, ethanol	Extraction	Application to damaged part	MMNB F117-168, P. 2

Propolis	External	Toothache	Propolis, ethanol	Extraction	Application to damaged part	VUBRS F81-921, P. 23–25

Propolis	External	Wounds	Propolis, ethanol	Extraction	Application to damaged part	LLTI BK III-343

Propolis	External	Joints pain	Propolis, sunflower oil	Solution in oil	Application to damaged part	LNMA FNM B172, P. 18

Propolis	Internal	Inside wounds	Propolis, ethanol	Extraction	Oral, drops in water	LNMA FNM B155, P. 8–10

Beeswax	External	Ear pain	Beeswax, linen cloth		Burning of rolled waxy linen cloth	LMDA I 1060 4/239

Beeswax	External	Abscess	Beeswax, sheep fat	Ointment	Application to damaged part	LLTI BK III-1154

Beeswax	External	Burned skin	Beeswax, egg		Application to damaged part	LLTI BK III-343

Beeswax	External	Abscess	Beeswax, butter, tuber of *Allium sativum *L.	Ointment	Application to damaged part	LKDA B6160, P. 19/74

Beeswax	External	Wounds	Beeswax, chicken fat, honey	Ointment	Application to damaged part	LMM, P. 155

Beeswax	External	“Rose” disease	Beeswax, fir resin, *Tilia cordata* Mill. flowers, *Matricaria recutita* L. flowers, butter	Ointment	Application to damaged part	MMA A418, P. 2

Beeswax	External	Infected wounds	Beeswax, tuber of *Allium sativum *L., lard, fir resin, soap	Ointment	Application to damaged part	MMNB, F127-87, P. 24

Beeswax	External	“Rose” disease	Beeswax, fir resin, lard	Ointment	Application to damaged part	LLTI BK III-1088
